# Source of Colors

**Published:** 1890-01

**Authors:** 


### Source of Colors.

The cochineal insects furnish a great many colors. Among them are carmine, crimson, scarlet carmine, and purple lakes. A sea shell belonging to the purpura, and found in Japanese waters, gives a rich violet dye. The cuttlefish gives the sepia. It is the inky fluid which the fish discharges in order to render the water opaque when attacked. Indian yellow comes from the feces of the camel. Ivory chips produce ivory black and bone black. Prussian blue is made by fusing horses' hoofs and other refuse animal matter with impure potassium carbonate. Various akes are derived from roots, barks and gums. Lamp black is soot from certain resinous substances. Turkey red is made from the madder plant, which grows in Hindostan. The yellow sap of a tree of Siam produces gamboge; the natives catch the sap in cocoanut shells. Raw sienna is the natural earth from the neighborhood of Sienna, Italy. Raw umber is also an earth found near Umbria and burnt. India ink is made from burnt camphor and gum. The Chinese and Japanese are the only manufacturers of this ink. The process is a tedious one and requires great skill. The finer grades of India ink are delicately scented with ottar of roses, and one stick about three inches long may cost four or five dollars. Age improves the ink. Mastic is made from the gum of the mastic tree, which grows in the Grecian Archipelago. Bistre is the soot of wood ashes. Very little real ultramarine is found in the market. It is obtained from the precious lapis-lazuli, and commands a fabulous price. Chinese white is zinc, scarlet is iodide of mercury, and native vermillion is from the quicksilver ore called cinnabar.

